# Use of Information Sources on Vaccine-Preventable Diseases in Pregnant Women: An Experience in Ferrara, Italy

**DOI:** 10.3390/ijerph17010233

**Published:** 2019-12-28

**Authors:** Giovanni Gabutti, Umberto Carioli, Diego Gamberoni, Giulia Masetti, Giulio Matteo, Paola Perrone, Rosaria Cappadona, Pantaleo Greco, Roberta Siliquini, Armando Stefanati

**Affiliations:** 1 Department of Medical Sciences, University of Ferrara, 44121 Ferrara, Italy; armando.stefanati@unife.it; 2 Postgraduate School of Hygiene and Preventive Medicine, University of Ferrara, 44121 Ferrara, Italydiego.gamberoni@unife.it (D.G.); giulia.masetti@unife.it (G.M.); giulio.matteo@unife.it (G.M.); paola.perrone@unife.it (P.P.); 3 Department of Morphology, Surgery and Experimental Medicine, Section of Obstetrics and Gynecology, Azienda Ospedaliero-Universitaria S. Anna, University of Ferrara, 44121 Ferrara, Italy; rosaria.cappadona@unife.it (R.C.); pantaleo.greco@unife.it (P.G.); 4 Department of Public Health Sciences and Pediatrics, University of Turin, 10126 Turin, Italy; roberta.siliquini@unito.it

**Keywords:** vaccine hesitancy, pregnancy, paediatric vaccination, vaccine

## Abstract

The “vaccine hesitancy” and the consequent lowering of vaccination coverage have, on one hand, pushed the Italian government to reintroduce some new compulsory vaccinations for access to schools and, on the other, have imposed a greater effort on health operators to understand the causes and, consequently, to intervene with tools for promotion and health education. In Ferrara, we administered 201 non-self-filling questionnaires to 201 pregnant women within a cross-sectional multicenter study, consisting of 63 items divided into 7 sections. In particular, we wanted to investigate the correlation between the socio-demographic characteristics of the interviewees and the sources used to obtain information and, on the other side, the intention to vaccinate in relation to the perception of the diffusion and of the risk of vaccine-preventable diseases. The institutional information sources are less used by foreigners, primiparous, and women with a low education level. The perception of the severity of vaccine-preventable diseases was greater in those inquiring from institutional sources. In a public health perspective, knowing the profile of future mothers in terms of socio-demographic characteristics and of the quality of the used information channels may help to guide the choices of communication in the vaccination field.

## 1. Introduction

Vaccines have allowed to prevent at least 10 million deaths between 2010 and 2015 [1] and the World Health Organization (WHO) estimates that immunization in the first decade of the 21st century prevented 2.5 million deaths every year in the world among children aged <5 years. Increasing immunization rates is an item on a list of ten public health achievements of the Center for Disease Control and Prevention (CDC) to improve life expectancy [2]. The Global Vaccine Action Plan 2011–2020 (GVAP), which was endorsed by all the WHO Member States at the 65th World Health Assembly in May 2012, described the immunization framework to prevent millions of deaths by 2020. The aim of GVAP 2011–2020 is also to guarantee the benefits of immunization, with a world perspective, to all people without any discrimination [3]. 

The European Vaccine Action Plan 2015–2020 (EVAP) attempts to reach GVAP’s goals in the WHO European Region. Unfortunately, the plan did not reach the targets and this area continues not be measles- and rubella-free [4].

The Italian document that acquires the European suggestions is the National Immunization Plan (Piano Nazionale Prevenzione Vaccinale, PNPV) 2017–2019, which recommends to keep immunization coverage in childhood at least at 95% against diphtheria, tetanus, pertussis, hepatitis B, *Haemophilus influenzae* type b, poliomyelitis, pneumococcus, meningococcus C, chickenpox, rotavirus, and the first dose of MMR (measles, mumps, and rubella) and HPV vaccine in both sexes at 12 years of age [5].

Considering the not optimal achievement of the coverage targets, their gradual decline in recent years, and the re-emerging outbreaks of measles [5,6,7], the Italian government introduced new mandatory vaccinations on 31 July 2017. In detail, the law 119/2017 [8] identifies ten mandatory vaccines for infants: diphtheria, tetanus, poliomyelitis, hepatitis B virus (all of which were already compulsory) plus pertussis, *Haemophilus influenzae* type b, measles, mumps, rubella, and varicella. Data related to the first six months since the application of this law showed an increasing immunization coverage for both mandatory and recommended immunizations [9].

Pregnant women play a key role because their opinion on and compliance to immunization indirectly affect the paediatric immunization coverage. A quite recent review suggests that parents have difficulties to identify impartial and reliable sources of information in order to obtain answers to their doubts on this specific topic [10].

One of the most common self-reported parent’s reasons that cause vaccination refuse or delay is the fear of vaccine-related adverse events and a low perception of infection’s risk related to vaccine-preventable diseases [11].

An experimental study points out that involving parents in focused meetings leads to a greater increase in paediatric vaccination if compared with self-reading information [12]. Furthermore, observational studies reveal that the web and health workers are the main sources of information used by pregnant women [13]. For this reason, there is an increasing attention to information available on the web, which anyway offers both misleading and evidence-based content [14].

In this context, efforts were made to create and improve institutional websites designed to spread scientific information on vaccines and immunization [15]. 

To better answer to health needs, it is necessary to overcome the concept of “vaccination consenting” or “refuser”. In 2012, the Strategic Advisory Group of Experts (SAGE) defined “vaccine hesitancy” as an attitude: “*to delay in acceptance or refusal of vaccination despite availability of vaccination services… influenced by factors such as complacency, convenience and confidence* “ [16].

The hesitant individuals are a heterogeneous group spreading between the range of total acceptance and categorical refusal. Within this continuum, those who are hesitant have a heterogeneous behavior. Gust D. et al. found that even if seriously concerned about vaccination parents are a low percentage, the remaining part, even if supportive, has some doubt about immunization safety [17].

In this perspective it is important to understand where and how people take information with the aim of spreading scientifically based information. A cross-sectional multicentre study (NAVIDAD study) examined the determinants of acceptance of mandatory vaccine restoration, identifying one of the main factors in information sources [18]. A subsequent analysis from the same working group focused on awareness and confidence in immunization [19]. In the context of the Navidad study, we analysed the use of information sources by pregnant women in the province of Ferrara.

## 2. Materials and Methods 

The study was approved by the Ethics Committee of Ferrara’s province (Ethical Project Identification Code: 160890), as part of a national cross-sectional multicentre study; the cities involved, in addition to Ferrara, were Turin (chief unit), Bologna, Milan, Parma, Ancona, Perugia, Roma, Siena, Catania, Chieti-Pescara, L’Aquila, Messina, and Naples.

During the period of November 2016–May 2017, we enrolled pregnant women, >18 years of age, who were asked to fill in a questionnaire. The women had to be able to understand the protocol information, in order to obtain an informed consent, and the questionnaire. Subjects of our sample were selected among patients waiting for a gynaecological visit, ultrasound, or other examinations at Ferrara’s University Hospital (which is the public Hospital of the city) and at the gynaecological counselling centre.

Before the interviews, we fully explained the aim of our study and, after that, we obtained the informed consent.

The interviews, lasting about 25 min, were conducted by trained resident doctors.

We administered a non-self-compiling questionnaire composed by seven sections for a total of 63 items [18]. Each section investigated the following topics:

1. Socio-demographic features (age, schooling, nationality, and so on);

2. Intention to vaccinate and for which pathologies;

3. Sources used to obtain informations about immunization;

4. The degree of confidence in healthcare workers;

5. The perception of the frequency and severity of the major vaccine-preventable diseases;

6. Vaccine knowledge;

7. The opinion on the restoration of mandatory vaccines for the admission to nursery and school.

This study focuses on the third section of the questionnaire, which investigates the sources through which the women had obtained information about vaccinations; in particular, we investigated how the choice of different kinds of sources (Section 3) is influenced by socio-demographic framing (Section 1) and how these sources influence the perception of the severity of the major vaccine-preventable diseases (Section 5).

A total of 201 questionnaires were collected and data were processed using IBM SPSS Statistics for Windows, Version 25.0. Armonk, NY: IBM Corp.. The variables included in the analysis were the following: socio-demographic data (previous delivery, marital status, nationality, education level, employment, age), the pregnancy quarter, the perception of severity of the major vaccine-preventable diseases (hepatitis B, poliomyelitis, *Haemophilus Influenzae* type B, tetanus, diphtheria, pertussis, measles, rubella, mumps, meningitis, varicella, and human papilloma virus), and the used information sources.

The perception of severity of the major vaccine-preventable diseases was classified as follows on the basis of the answer to the question, “how much do you consider dangerous the following disease?”: severe (including the answers in the questionnaire “very severe” and “quite severe”) and not severe (including the answers “not severe at all”, “a little severe”, and “no answer”).

The sources used by women to seek and obtain information about vaccinations were classified in “institutional sources” (general practitioner, gynaecologist, paediatrician, information leaflets from the Ministry of Health or the healthcare services, vaccination clinics, institutional websites, birthing classes) and “not institutional” (not institutional websites, applications for smartphone or tablet, clinicians not working for public health service, word of mouth through relatives and friends, mass media, anti-vaccine movements). We divided our sample into “women who received information from at least two institutional sources” and “women who received one or none information from institutional sources” in order to investigate, by a bivariate analysis, the association between the used information source and the perception of severity of the vaccine-preventable diseases.

A multivariate logistic regression model has been estimated, considering the source of information (institutional or not institutional) as the dependent variable and the socio-demographic variables as covariates (previous delivery, marital status, nationality, education level, employment, quarter of pregnancy and age). About delivery we consider the difference between women who had at least one previous delivery and nulliparity; about education between “high level” (if they had a college degree or high school), and “low level” (if they have a lower degree than high school); about employment we consider the difference between unemployed and employed; about the age we divided the sample around the average age of the Italian pregnant women (33 years) reported by the CEDAP (Certificato di assistenza al parto/*Birth attendance certificate*) [20]. All variables investigated in the socio-demographic part of the questionnaire were included in the logistic regression model, independently from its statistical significance.

These associations are expressed as odds ratios (OR) with a 95% confidence interval (CI), a *p*-value ≤ 0.05 was considered significant for all analyses.

## 3. Results

A total of 201 pregnant women were interviewed, and their filled in questionnaires were collected.

The socio-demographic characteristics of the sample are described in Table 1.

The average age of the sample was equal to 33.5 years (32.6 years for primiparous); only 6.5% of women were aged 25 or under, while 10% were >40 years old. A total of 72.6% (146) of the respondents faced their first pregnancy. Only 7% were foreigners, and 190 (94.5%) were married or life-partners.

Regarding the educational qualification, 95 women (47.3%) were graduates, 87 (43.3%) had a high school degree, and 19 (9.5%) had a lower degree.

Most of the interviewed women were in the third quarter of pregnancy (68.2%) as most questionnaires were administered in outpatient facilities, where there were also places for pre-natal courses, or in clinics dedicated to term pregnancies.

20.4% of the sample was composed by unemployed women; most (42.3%) were office employers or teachers, 13.4% were healthcare workers.

The use of institutional information sources concerning vaccination is shown in Table 2. 

Women showing a lesser probability to use institutional sources are primiparous (Adj OR: 0.042, 95% CI: 0.014–0.122, *p* < 0.001), foreign (Adj OR: 0.213, 95% CI: 0.046–0.980, *p* = 0.047), and with a low level of education (Adj OR: 0.197, 95% CI: 0.049–0.790, *p* = 0.022). Women ≥33 years of age and women who were in the third quarter at the time of the interview showed a greater tendency to use institutional sources, although these data have no statistical significance.

Table 3 shows how the perception of risk related to the main vaccine-preventable diseases is influenced by the type of used source of information: institutional or non-institutional. The analysis underlines that the perception of the risk related to vaccine-preventable diseases is overall greater among women using institutional sources to obtain information. In detail, it emerges that, for poliomyelitis, *Haemophilus influentiae* type b, diphtheria, pertussis, and rubella, the difference between the two groups of women is statistically significant.

## 4. Discussion

In this historical moment of reluctance to immunization, the quality of information sources plays a pivotal role in supporting trust in healthcare workers and health systems.

Investing in information sources could have a positive impact. For this reason, in our analysis, we wanted to investigate the characteristics of those who use institutional information sources and how these later influence risk perception.

In fact, as previously described in Table 2, we found that some socio-demographic characteristics are more strongly associated with a poor use of institutional information sources; in particular, we found that nulliparity is statistically very rarely associated with the use of institutional information sources compared with those who have already had at least one pregnancy.

Women in the first pregnancy have not yet experienced the search for information; we can suppose that in this phase the woman is more concentrated on the pregnancy itself and on the imminent delivery than on future vaccinations. Noteworthy, accordingly to the literature, mother’s hesitancy decreases during the first two years after child’s birth for an increased maternal experience about vaccinations [21]. 

The interviewed foreign women, representing 7% of our sample, had a lower probability of using institutional sources than the Italian ones; maybe the cultural barrier makes less easy to find information and entails a probable lower inclination to access to health care services.

In the literature, the impact of education level on immunization coverage is ambiguous [22,23,24]. In our case, we found that low schooling is associated with less research for information from institutional sources compared to those who have at least a high school diploma. 

Regarding the perception of the diseases’ danger, we found that this is significantly related to the quality of the information sources. A similar analysis has been conducted and it has shown that the sources of information influence the level of knowledge of the pregnant women [19].

Regarding single vaccine-preventable diseases, we found statistically significant association only for poliomyelitis, *Haemophilus influentiae* type b, diphtheria, pertussis and rubella. It is worrying that, in both groups of the population, some diseases, still very widespread such as measles, have not given significant results. This phenomenon may perhaps be attributed to the fact that some diseases are linked to a personal experience of low severity such as measles and chickenpox. But, on the other hand, poliomyelitis and diphtheria still frighten women despite being almost eliminated/eradicated. Poliomyelitis in fact still has a high media echo despite its low spread. The lack of statistical significance associated with the perception of dangerousness of meningitis between the two groups, could be attributed on one hand, to the media’s echo that it has had in recent years. In fact, as happens during epidemic events, even during the recent meningitis emergency in Tuscany [25,26], it led health authorities to use also the mass media as institutional channels (which in our study were classified as “not institutional”) for their rapidity and capillarity to reach the population. On the other, it could be attributed to the intrinsic high outrage level of meningococcal diseases, as recently evidenced [27].

## 5. Conclusions

Women, and mostly when they are pregnant, are sensitive subjects to the issue of vaccination as they are more vigilant to the health of their children. During pregnancy it is important to take advantage of the frequent contact the women have with health professionals to convey correct information. This assumes that health professionals must be aware of factors influencing immunization compliance. In fact, knowing the socio-demographic characteristics of future mothers and the quality of the used information channels may guide to the correct choice of communication. In the future is desirable that health authorities increase also the use of non-institutional sources to convey evidence-based information in order to reach the majority of people.

## Figures and Tables

**Table 1:** Socio-demographic characteristics of the sample.

		*n*	%
Age (years)	≤20	4	2.0
	21–25	9	4.5
	26–30	50	24.9
	31–35	60	29.9
	36–40	58	28.9
	>40	20	10.0
Previous delivery	One or more	55	27.4
	None	146	72.6
Nationality	Italian	187	93.0
	Foreign	14	7.0
Marital status	Cohabiting/married	190	94.5
	Single/divorced	11	5.5
Education level	College degree	95	47.3
	High school	87	43.3
	Lower than high school	19	9.5
Quarter of pregnancy	First	26	12.9
	Second	38	18.9
	Third	137	68.2
Employment	Office workers or teachers	85	42.3
	Entrepreneurs or freelance professionals	18	9.0
	Labourers or artisans	19	9.5
	Healthcare professional	27	13.4
	Unemployed	41	20.4
	Other	11	5.5

**Table 2:** Association between socio-demographic data and vaccine information from institutional sources. OR, odds ratio; CI, confidence interval.

		Vaccination Information from Two Institutional Sources at least					
		OR	95% CI	*p* **	Adj OR *	95% CI	p **
Previous delivery	One or more	1000			1.000		
	**None**	**0.078**	**0.033–0.186**	**<0.001**	**0.042**	**0.014–0.122**	**<0.001**
Marital status	Cohabiting/married	1000			1.000		
	Single/divorced	0.367	0.095–1.426	0.148	0.336	0.069–1.634	0.176
Nationality	Italian	1000			1.000		
	**Foreign**	0.489	0.161–1.487	0.208	**0.213**	**0.046–0.980**	**0.047**
Education level	High level	1000			1.000		
	**Low level**	0.571	0.215–1.515	0.260	**0.197**	**0.049–0.790**	**0.022**
Employment	Unemployed	1000			1.000		
	Employed	0.906	0.456–1.800	0.778	0.604	0.250–1.457	0.262
Quarter of pregnancy	First or second	1000			1.000		
	Third	1261	0.695–2.287	0.445	1.769	0.824–3.796	0.143
Age (years)	<33	1,000			1.000		
	≥33	1,271	0.722–2.237	0.405	1.062	0.535–2.106	0.864

Statistically significant results are reported in bold. * Adjusted age, marital status, nationality, educational level, employment, quarter of pregnancy, previous delivery. ** Significance level *p* < 0.05.

**Table 3:** Perception of risk related to vaccine-preventable infectious disease according to the type of information source: institutional or non-institutional.

	Disease Dangerousness Perception	Vaccination Information from two Institutional Sources at least					
		Yes		No			
		*n* (=99)	%	*n* (=102)	%	OR	*p* *
Hepatitis B	Yes	94	50.5	92	49.5	1.644	0.200
	No	5	33.3	10	66.7		
Poliomyelitis	Yes	92	53.2	81	46.8	7.656	**0.006**
	No	7	25.0	21	75.0		
*Haemophilus influenzae* b	Yes	40	67.8	19	32.2	11.489	**0.001**
	No	59	41.5	83	58.5		
Tetanus	Yes	90	50.8	87	49.2	1.506	0.220
	No	9	37.5	15	62.5		
Diphtheria	Yes	74	54.4	62	45.6	4.477	**0.034**
	No	25	38.5	40	61.5		
Pertussis	Yes	75	54.7	62	45.3	5.190	**0.023**
	No	24	37.5	40	62.5		
Measles	Yes	72	53.3	63	46.7	2.738	0.098
	No	27	40.9	39	59.1		
Rubella	Yes	76	54.7	63	45.3	5.301	**0.021**
	No	23	37.1	39	62.9		
Mumps	Yes	61	51.7	57	48.3	0.681	0.409
	No	38	45.8	45	54.2		
Meningitis	Yes	98	50.5	96	49.5	3.548	0.060
	No	1	14.3	6	85.7		
Varicella	Yes	55	52.4	50	47.6	0.860	0.354
	No	44	45.8	52	54.2		
HPV	Yes	55	52.4	50	47.6	0.860	0.354
	No	44	45.8	52	54.2		

Statistically significant results are reported in bold. * Chi-squared test, significance level *p* < 0.05.
